# Early goal-directed and lactate-guided therapy in adult patients with severe sepsis and septic shock: a meta-analysis of randomized controlled trials

**DOI:** 10.1186/s12967-018-1700-7

**Published:** 2018-11-29

**Authors:** Xian-Fei Ding, Zi-Yue Yang, Zhen-Tao Xu, Li-Feng Li, Bo Yuan, Li-Na Guo, Le-Xin Wang, Xi Zhu, Tong-Wen Sun

**Affiliations:** 1 General ICU, The First Affiliated Hospital of Zhengzhou University, Zhengzhou, 450052 China; 2 Henan Key Laboratory of Critical Care Medicine, Zhengzhou, 450052 China; 3 Department of Pharmacy, The First Affiliated Hospital of Zhengzhou University, Zhengzhou, 450052 China; 4 Biotherapy Center, The First Affiliated Hospital of Zhengzhou University, Zhengzhou, 450052 China; 5 Department of Neurology, The First Affiliated Hospital of Zhengzhou University, Zhengzhou, 450052 China; 6 School of Biomedical Sciences, Charles Sturt University, Wagga, 2650 Australia; 7 Department of Critical Care Medicine, Peking University Third Hospital, Beijing, 100191 China

**Keywords:** EGDT, Lactate-guided therapy, Usual care, Sepsis, Meta-analysis

## Abstract

**Background:**

The ProCESS, ARISE, and ProMISe trials have failed to show that early goal-directed therapy (EGDT) reduces mortality in patients with severe sepsis and septic shock. Although lactate-guided therapy (LGT) has been shown to result in significantly lower mortality, its use remains controversial. Therefore, we performed a meta-analysis to evaluate EGDT vs. LGT or usual care (UC) in adult patients with severe sepsis and septic shock.

**Methods:**

Relevant randomized controlled trials published from January 1, 2001 to March 30, 2017 were identified in PubMed, EMBASE, Web of Science, and the Cochrane Library. The primary outcome was mortality; secondary outcomes included red cell transfusions, dobutamine use, vasopressor infusion, and mechanical ventilation support within the first 6 h and Acute Physiology and Chronic Health Evaluation II (APACHE II) score.

**Results:**

Sixteen studies enrolling 5968 patients with 2956 in EGDT, 2547 in UC, and 465 in LGT were included in this meta-analysis. Compared with UC, EGDT was associated with a lower mortality (10 trials; RR 0.85, 95% CI 0.74–0.97, *P* = 0.01), and this difference was more pronounced in the subgroup of UC patients with mortality > 30%. In addition, EGDT patients received more red cell transfusions, dobutamine, and vasopressor infusions within the first 6 h. Compared with LGT, EGDT was associated with higher mortality (6 trials; RR 1.42, 95% CI 1.19–1.70, *P* = 0.0001) with no heterogeneity (*P* = 0.727, *I*^2^ = 0%).

**Conclusion:**

EGDT seems to reduce mortality in adult patients with severe sepsis and septic shock, and the benefit may primarily be attributed to red cell transfusions, dobutamine administration, and vasopressor infusions within the first 6 h. However, LGT may result in a greater mortality benefit than EGDT.

**Electronic supplementary material:**

The online version of this article (10.1186/s12967-018-1700-7) contains supplementary material, which is available to authorized users.

## Background

Sepsis is a physiological, pathological, and biochemical syndrome induced by infection and is a major public health concern, accounting for more than $20 billion (5.2%) of total US hospital costs in 2011 [1]. Although its true incidence is unknown, conservative estimates indicate that sepsis is a leading cause of mortality and critical illness worldwide [2, 3].

In 2001, Rivers et al. [4] conducted a 263-patient, single-centre RCT comparing early goal-directed therapy (EGDT) with usual care (UC) in patients with septic shock visiting an urban emergency department (ED) in the US. EGDT was provided through a 6-h resuscitation protocol that involved the administration of intravenous fluids, vasopressors, inotropes, and red cell transfusions to achieve pre-specified targets for arterial blood pressure, central venous pressure, systemic central venous oxygen saturation (Scvo_2_), and haemoglobin level. EGDT was found to reduce hospital mortality from 46.5 to 30.5% [4], prompting its adoption by many institutions worldwide [5]. Subsequently, three large multicentre RCTs, namely, the ProCESS [6], ARISE [7], and ProMISe [8] trials, failed to show that EGDT reduced mortality to a greater extent than UC in patients with severe sepsis and septic shock; however, no harm was associated with the intervention strategies. Of note, the patients in these trials [6–8] were less severely ill (lower baseline lactate levels, Scvo_2_ at or above the target value on admission, and lower mortality in the control group). Although EGDT cannot currently be recommended with the available evidence base, bedside clinicians still need guidance regarding how to approach this group of patients, who have significant mortality and morbidity [9], and the use of EGDT is still safe and may be considered.

Lactate is a standard laboratory parameter measured with prescribed techniques and may serve as a more objective surrogate for tissue perfusion than physical examination or urine output. Additionally, we know that increased lactate levels are associated with worse outcomes [10]. Lactate clearance, defined by the change in lactate levels between 2 points in time, is a much more rapid, less invasive and less costly measurement than physical examination [11, 12]. Lactate clearance and Scvo_2_ can be used as markers of early recovery of fluid targets, as the lactate clearance rate has been found to be an accurate and reliable indicator of fluid status [13]. In 2004, lactate clearance was shown to be comparable to Scvo_2_ as a perfusion marker and may therefore function as an instructional target in early resuscitation for septic shock [11]. Accordingly, 6 RCTs have shown significantly lower mortality with LGT resuscitation versus other protocols [13–18]. In addition, several studies have suggested that a significant degree of technical difficulty is associated with the use of computerized spectrophotometric catheters to simultaneously monitor Scvo_2_. Using Scvo_2_ requires preplanned training and real-time calibration, and it may be limited by time, expertise, and the need for specialized equipment [19]. Therefore, the prospect of monitoring lactate clearance has gradually attracted interest, and the Surviving Sepsis Campaign (SSC) 2016 also suggested guiding resuscitation by normalizing lactate in patients with elevated lactate levels as a marker of tissue hypoperfusion [9]. However, the use of LGT to guide resuscitation remains controversial.

Previous meta-analyses [20–23] of EGDT vs. UC did not assess studies with interventions that included red cell transfusion, dobutamine use, vasopressor infusion, and mechanical ventilation support within the first 6 h and Acute Physiology and Chronic Health Evaluation II (APACHE II) scores. Therefore, we conducted this meta-analysis to evaluate the effect of EGDT compared with LGT or UC on mortality among adults with severe sepsis and septic shock.

## Methods

We conducted a meta-analysis using the guidelines recommended by the Cochrane Collaboration in the Cochrane Handbook for Systematic Reviews of Interventions (http://www.cochranehandbook.org). Our meta-analysis was conducted and reported based on the Preferred Reporting Items for Systematic Reviews and Meta-Analyses (PRISMA) guidelines [24]. The PRISMA 2009 checklist is shown in Additional file 1.

### Search strategy

**Fig. 1:**
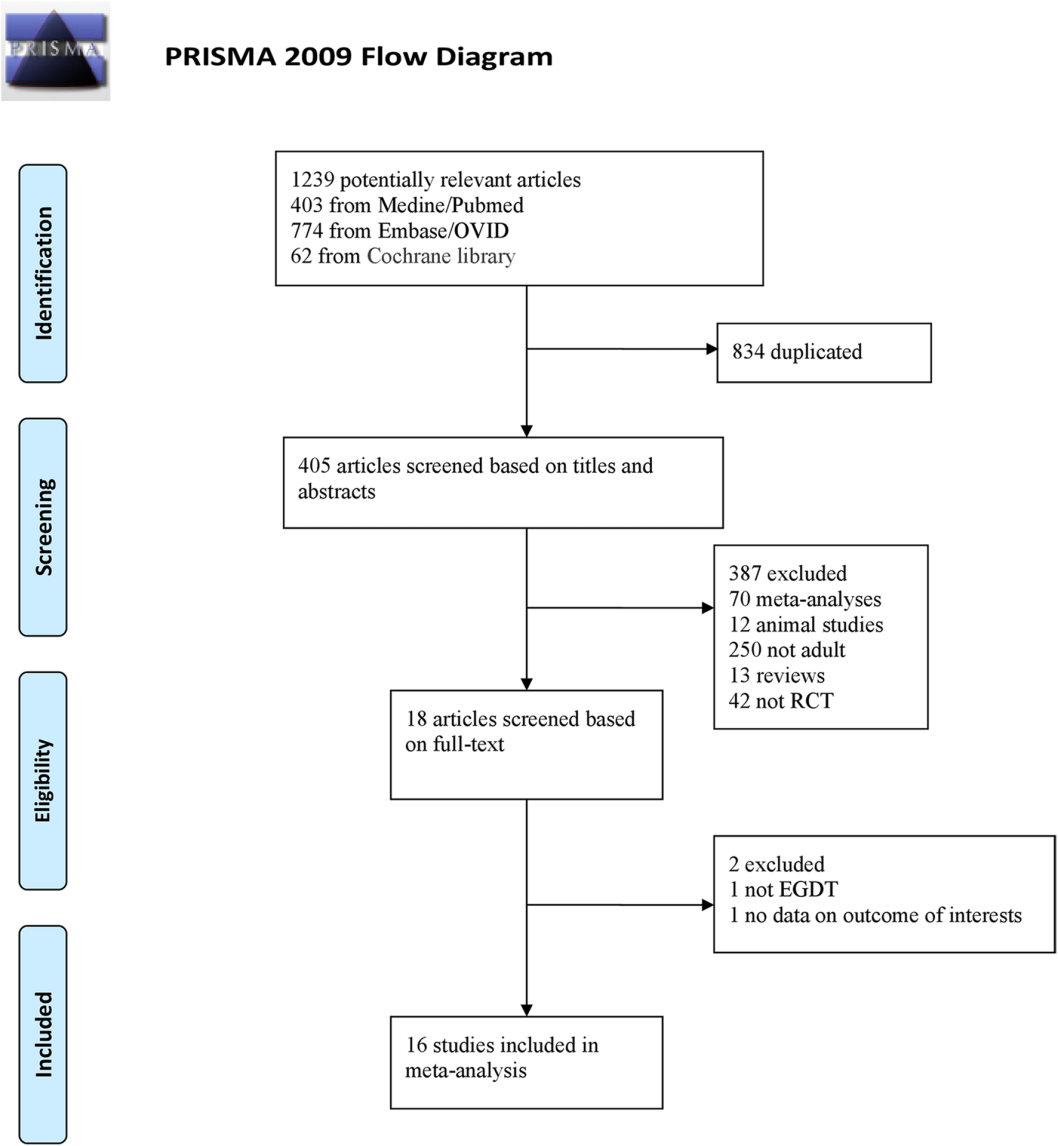
Flow chart for the study selection

The PubMed, EMBASE, ISI Web of Science, and Cochrane Library databases were searched systematically to identify all RCTs published from January 1, 2001, to March 30, 2017, assessing EGDT in adult patients with severe sepsis and septic shock. The search process is shown in Fig. 1. The following terms were used: “Early goal-directed therapy” or “EGDT”, “lactate”, and “sepsis” or “septic shock” or “severe sepsis”. There were no language restrictions.

### Study selection

Two authors independently checked the title and abstract of the studies to determine whether they potentially met the inclusion criteria. We also recorded the reasons for excluding trials. We resolved disagreements through discussion.

### Inclusion criteria

#### Patients

Relevant RCT studies compared EGDT with LGT or UC in adults with severe sepsis and septic shock.

#### Interventions

Standard EGDT was defined as outlined in the SSC, namely, achieving central venous pressure of 8 to 12 mmHg, mean arterial pressure of 65 to 90 mmHg, urine output of 0.5 ml/kg/h or more, and Scvo_2_ of 70% or above within the first 6 h of intervention [25].

#### Comparison

Control care, including UC (UC was defined as conventional treatment, which was at the discretion of the clinicians) and LGT (lactate or lactate clearance monitoring with the aim to decrease levels), was used for comparison.

#### Outcomes

The primary endpoint was all-cause mortality. When several mortality rates were reported in one study, we used the primary outcome. Secondary outcomes included red cell transfusion, dobutamine use, vasopressor infusion, mechanical ventilation support within the first 6 h, and APACHE II scores.

#### Study design

We included all RCTs comparing EGDT with UC or LGT in patients with severe sepsis or septic shock.

#### Exclusion criteria

The exclusion criteria were as follows: (1) studies with duplicated data; (2) non-randomized studies; (3) meta-analyses, animal studies, studies of non-adult populations, or reviews; and (4) studies published before January 2001.

### Data extraction

Two authors independently extracted the data from each trial, including the name of the first author, year of publication, demographic characteristics (population, country, clinical setting), centre, number of patients, mortality, study design, and APACHE II score.

### Risk of bias

Reviewers independently assessed the risk of bias using the Cochrane collaboration tool [24]. Two authors respectively rated the risks (as “low”, “unclear” or “high” risk of bias) regarding 7 domains: random sequence generation, allocation concealment, blinding of participants and personnel, blinding of outcome assessment, incomplete outcome data, selective reporting, and other bias. High risk of bias in any one or more of the key domains of research indicated that the study had a high risk of bias. Studies with a low risk of bias in all key domains were considered to have a low risk of bias overall. All other studies were considered to have an unclear risk of bias.

### Statistical analysis

For dichotomous data, we estimated the relative risk (RR) and 95% confidence interval (CI) to describe the size of the treatment effect. We performed all analyses using the intention-to-treat principle and generated funnel plots to evaluate publication bias. We quantified statistical heterogeneity using the *I*^2^ statistic, calculated as *I*^2^ = 100% ×  (Q − *df*)/Q, where Q is Cochran’s heterogeneity statistic [26]. *I*^2^ values of 0–24.9% indicated no heterogeneity, 25–49.9% indicated mild heterogeneity, 50–74.9% indicated moderate heterogeneity, and 75–100% indicated considerable heterogeneity. The pooled RR of each study was calculated using the fixed-effects model (Mantel–Haenszel) or random-effects model (DerSimonian and Laird). All reported *P* values were two-sided, and *P* values < 0.05 were considered to indicate statistically significant differences. All analyses were performed using Review Manager Software (RevMan, version 5.3).

## Results

### Literature search

The PRISMA statement flowchart shows the literature screening process, study selection, and reasons for exclusion, as presented in Fig. 1. The literature search yielded 1239 potentially relevant articles. We removed 834 duplicate studies based on title screening, and after the abstract evaluation, 387 studies were excluded for not meeting the inclusion criteria. After the full texts were reviewed, a total of 16 RCTs [4, 6–8, 13–18, 27–32] (n = 5968) were finally included in this meta-analysis. There was 100% agreement between the 2 reviewers regarding study inclusion and exclusion.

### Study characteristics

The main characteristics extracted from the 16 RCTs [4, 6–8, 13–18, 27–32] are shown in Table 1. This meta-analysis enrolled 5968 patients:2956 in the EGDT, 2547 in the UC, and 465 in the LGT groups. These trials were published from January 1, 2001, to March 30, 2017, with sample sizes ranging from 33 to 1588. Of the 16 trials, 6 [6, 7, 14–16, 27] were multi-centre studies, and the others [4, 8, 13, 17, 18, 24–26, 28–32] were single-centre studies. Ten trials [4, 6–8, 27–32] reported EGDT vs. UC, and 6 trials [13–18] reported EGDT vs. LGT. There were 3 trials [4, 6, 15] from the USA, 1 trial [32] from Taiwan, 1 trial [28] from Zambia, 1 trial [7] from Australasia/New Zealand, 1 trial [8] from England, 1 trial [14] from the Netherlands, and 8 trials [13, 16–18, 27, 29, 30, 32] from China. Ten trials [13, 14, 16–18, 27, 29–32] were conducted in the intensive care unit (ICU), 4 trials [4, 7, 15, 16] were conducted in the ED, and the remaining trials were conducted in the ICU or ED.

**Table 1:** Patient characteristics and primary mortality outcome of the included studies

Study	Arms	Demographic characteristics				Number of patients		Mortality		Primary outcome (mortality)	Study design
		Population	Country	Clinical setting	Centre	EGDT	UC/LGT	EGDT (%)	UC/LGT (%)		
Rivers 2001 [4]	EGDT/UC	Adult	USA	ED	S	130	133	29.2	44.5	In-hospital	RCT
Lin 2006 [31]	EGDT/UC	Adult	Taiwan	ICU	S	108	116	53.7	71.6	In-hospital	RCT
Wang 2006 [32]	EGDT/UC	Adult	China	ICU	S	16	17	25.0	41.2	14-day	RCT
Chen 2007 [29]	EGDT/UC	Adult	China	ICU	S	88	102	29.5	49.0	In-hospital	RCT
He 2007 [30]	EGDT/UC	Adult	China	ICU	S	98	105	52.0	64.8	In-hospital	RCT
Yan 2010 [27]	EGDT/UC	Adult	China	ICU	M	157	146	35.0	50.7	28-day	RCT
Andrews 2014 [28]	EGDT/UC	Adult	Zambia	ED/ICU	S	53	56	64.2	60.7	In-hospital	RCT
ARISE 2014 [7]	EGDT/UC	Adult	Australasia/New Zealand	ED	M	792	796	18.6	18.8	90-day	RCT
ProCESS 2014 [6]	EGDT/UC	Adult	USA	ED/ICU	M	439	456	21.0	18.9	60-day	RCT
ProMISe 2015 [8]	EGDT/UC	Adult	England	ED	M	623	620	29.5	29.2	90-day	RCT
Jansen 2010 [14]	EGDT/LGT	Adult	Netherlands	ICU	M	177	171	43.5	33.9	In-hospital	RCT
Jones 2010 [15]	EGDT/LGT	Adult	USA	ED	M	150	150	23.0	17.0	In-hospital	RCT
Tian 2012 [17]	EGDT/LGT	Adult	China	ICU	S	19	43	63.2	32.6	28-day	RCT
Yu 2013 [13]	EGDT/LGT	Adult	China	ICU	S	25	25	28.0	20.0	28-day	RCT
Wang 2014 [18]	EGDT/LGT	Adult	China	ICU	S	31	26	54.8	26.9	28-day	RCT
Lv 2015 [16]	EGDT/LGT	Adult	China	ICU	S	50	50	56.0	40.0	28-day	RCT

RCT, randomized controlled trial; EGDT, early goal-directed therapy; UC, usual care; LGT, lactate-guided therapy; ED, emergency department; ICU, intensive care unit; S, single-centre; M, multi-centre

### Risk of bias and quality of evidence

**Fig. 2:**
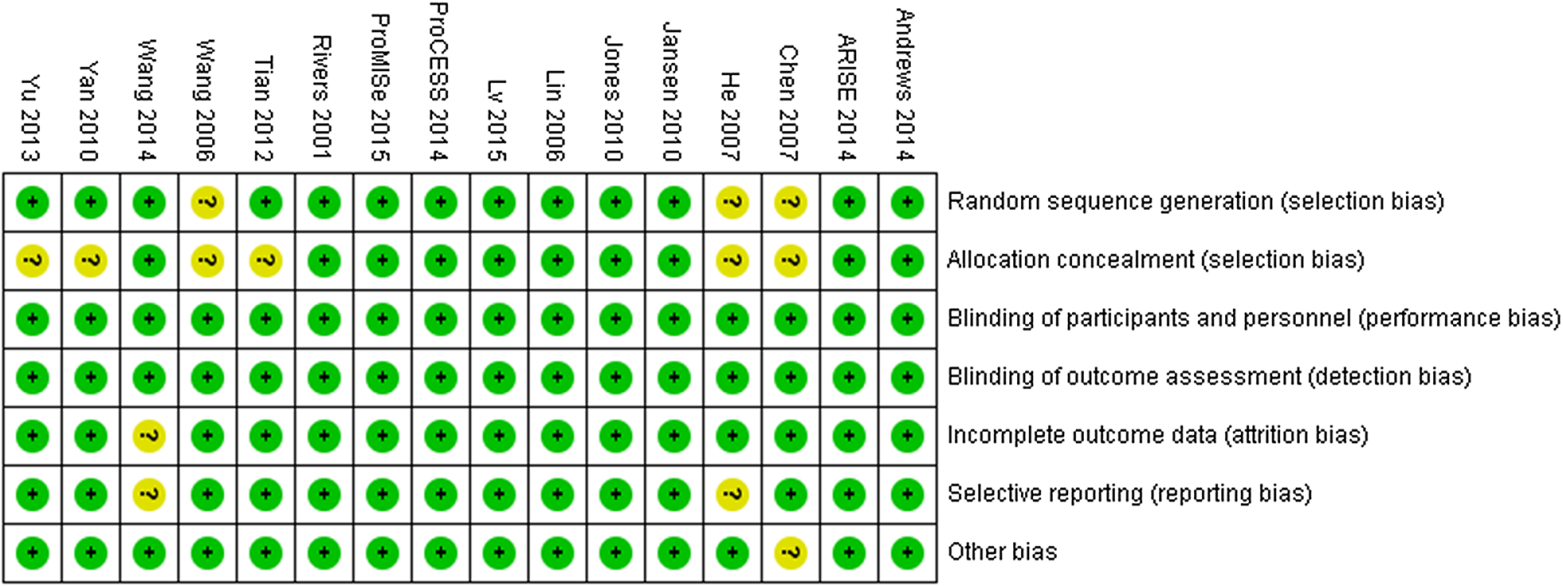
Risk of bias summary reviewing authors’ evaluations of each risk of bias item for each included study. Green circles indicate low risk of bias, yellow circles indicate unclear risk of bias, and red circles indicate high risk of bias

The assessment of risk of bias is shown in Fig. 2. Nine trials [4, 6–8, 14–16, 28, 31] were considered to have a low risk of bias, and the remaining 7 trials [17, 18, 25, 27, 29, 30, 32] had an unclear risk of bias. All trials were RCTs. However, blinding of patients and clinicians to evaluate a complex intervention such as EGDT is extremely difficult, and the authors concluded that the primary outcome was unlikely to be influenced by the lack of blinding.

### Primary outcome

**Fig. 3:**
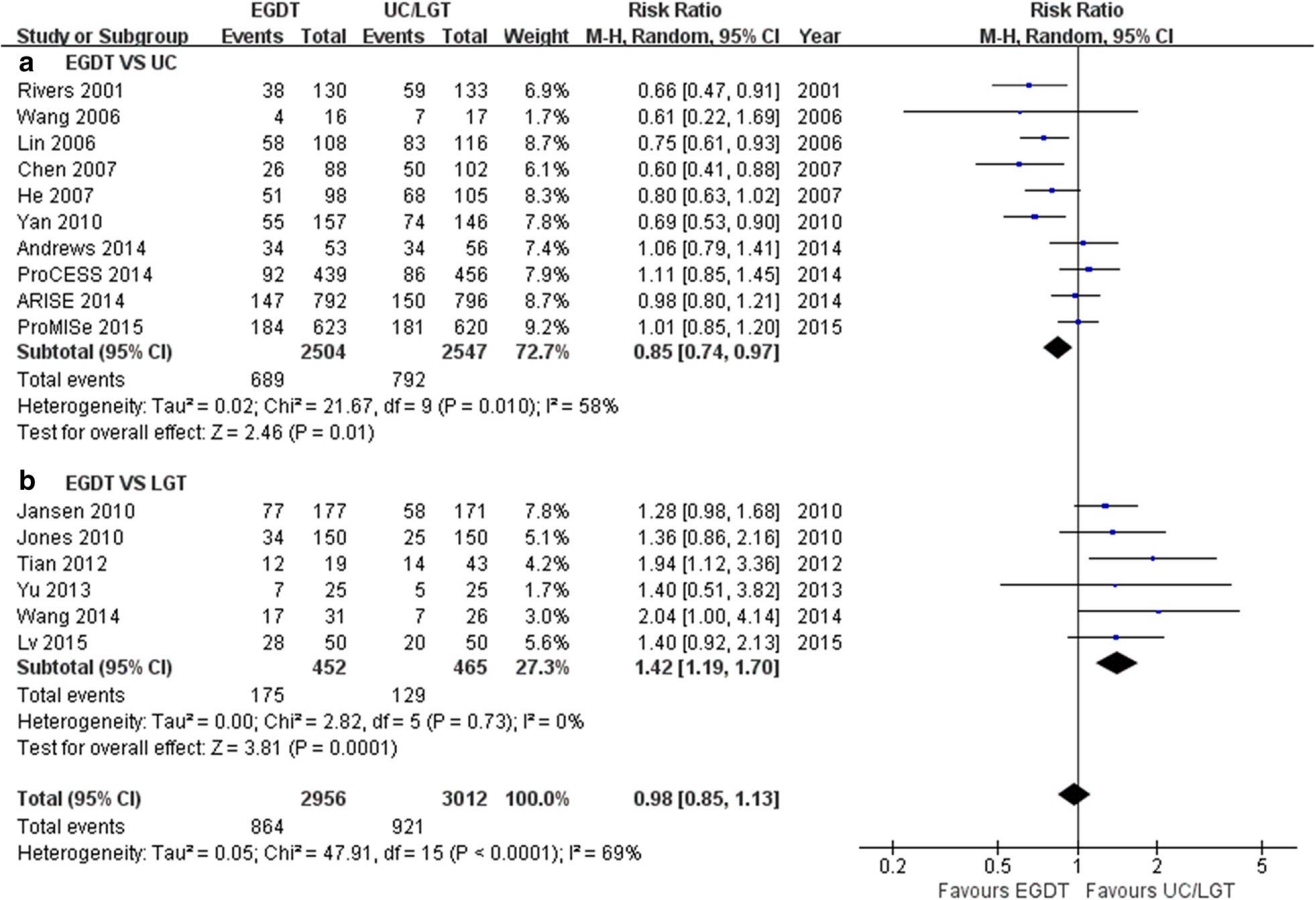
Forest plot showing the effect of EGDT vs. UC/LGT on mortality in patients with severe sepsis and septic shock. The analysis was stratified by UC or LGT. RR < 1.0 favours EGDT

Due to the moderate heterogeneity (*I*^2^ = 69%, *P *< 0.0001) of the pooled RR from 16 trials, a random-effects model was used for the meta-analysis of EGDT versus (UC + LGT). Ten trials [4, 6–8, 27–32] containing 5051 patients compared EGDT with UC in terms of all-cause mortality. Compared with UC, EGDT was associated with lower mortality (RR 0.85, 95% CI 0.74–0.97, *P* = 0.01; *I*^2^ = 58.5%, *P* = 0.010; Fig. 3). Six trials [13–18] including 917 patients compared the mortality of EGDT with that of LGT. Compared with LGT, EGDT was associated with higher mortality, with no heterogeneity (RR 1.42, 95% CI 1.19-1.70, *P* = 0.0001; *I*^2^ = 0%, *P* = 0.73; Fig. 3).

**Fig. 4:**
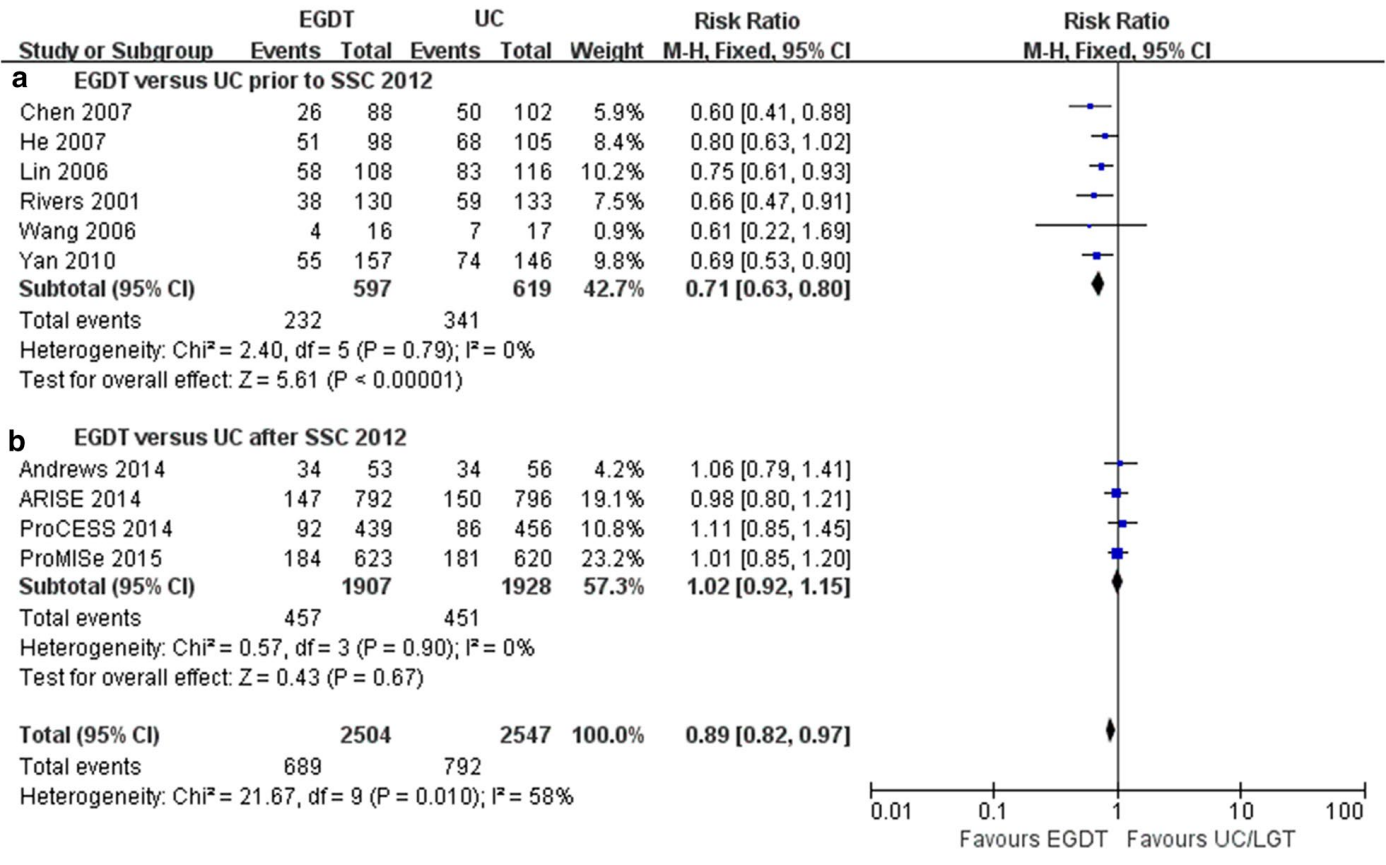
Forest plot showing EGDT vs. UC regarding mortality in patients with severe sepsis and septic shock. The analysis was stratified by publication year in relation to the SSC 2012 for UC. RR < 1.0 favours EGDT

Subgroup analyses according to publication year showed that the mortality benefit of EGDT was observed only in the subgroup of RCTs published prior to the SSC2012 (6 trials; RR = 0.71, 95% CI 0.63–0.80, *P *< 0.0001; *I*^2^ = 0%, *P* = 0.79) [4, 27, 29–32] and not in the subgroup of RCTs published after the SSC 2012 by the fixed-effects model (4 trials; RR = 1.02, 95% CI 0.92–1.15, *P *= 0.67; *I*^2^ = 0%, *P* = 0.90) [6–8, 28] (Fig. 4).

**Fig. 5:**
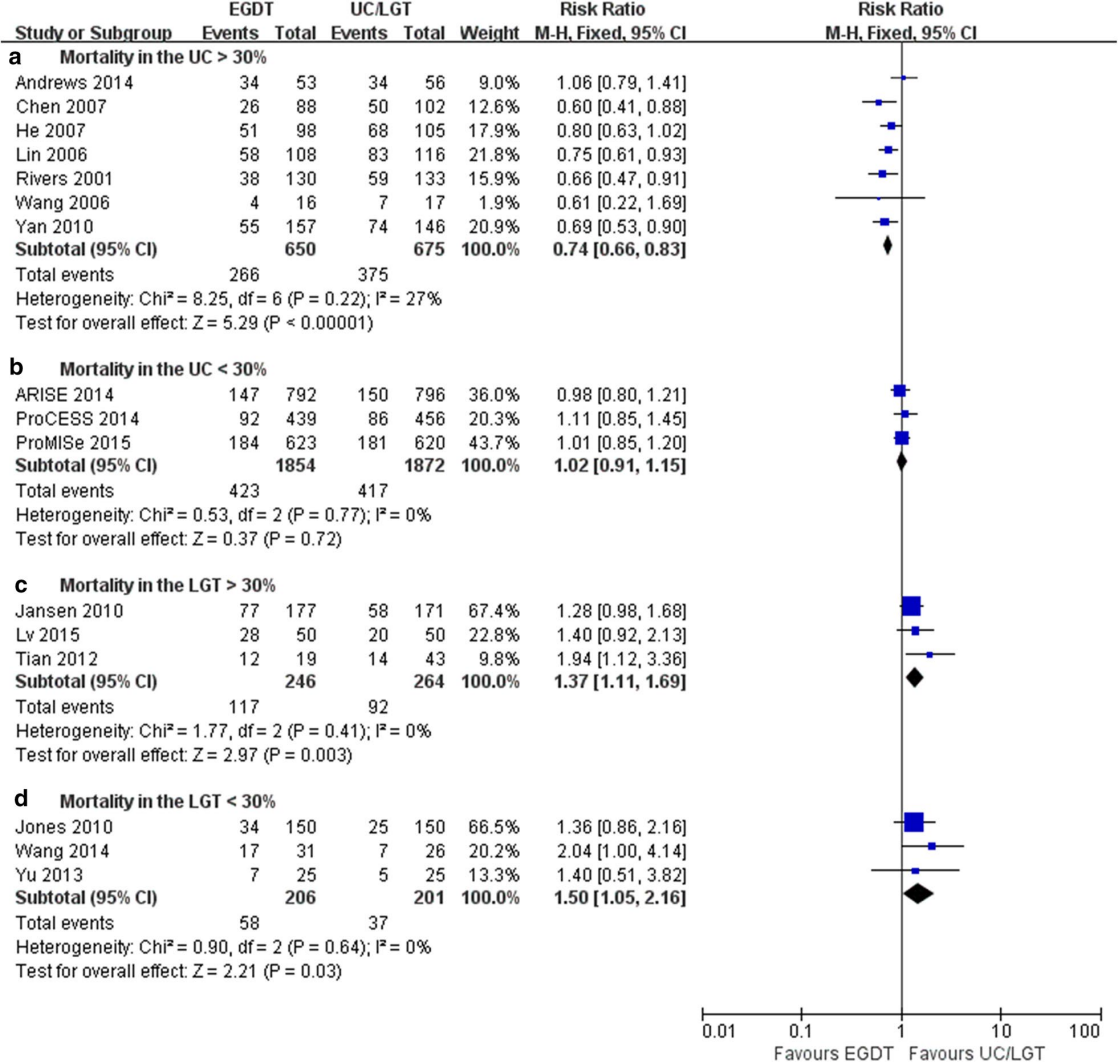
Forest plot showing EGDT vs. UC/LGT regarding mortality in patients with severe sepsis and septic shock. The analysis was stratified by UC or LGT, mortality > or < 30%. RR < 1.0 favours EGDT

Further subgroup analyses revealed that EGDT had a lower mortality than UC when the mortality of UC was > 30% (7 trials; RR = 0.74, 95% CI 0.66–0.83, *P *< 0.0001; *I*^2^ = 27%, *P* = 0.22) [4, 27–32] but not when the mortality of UC was < 30% (3 trials; RR = 1.02, 95% CI 0.91–1.15, *P* = 0.72; *I*^2^ = 0%, *P* = 0.77) [6–8]. In addition, EGDT resulted in higher mortality than LGT when the mortality of the LGT group was both > and < 30% (3 trials, RR = 1.37, 95% CI 1.11–1.69, *P *= 0.003; *I*^2^ = 0%, *P* = 0.41 [14, 16, 17] and 3 trials, RR = 1.50, 95% CI 1.05–2.16, *P *= 0.003; *I*^2^ = 0%, *P* = 0.64 [13, 15, 18], respectively) (Fig. 5). Due to the low heterogeneity in the subgroup analyses of the mortality, the fixed-effects model was used for the meta-analysis.

### Secondary outcomes

**Fig. 6:**
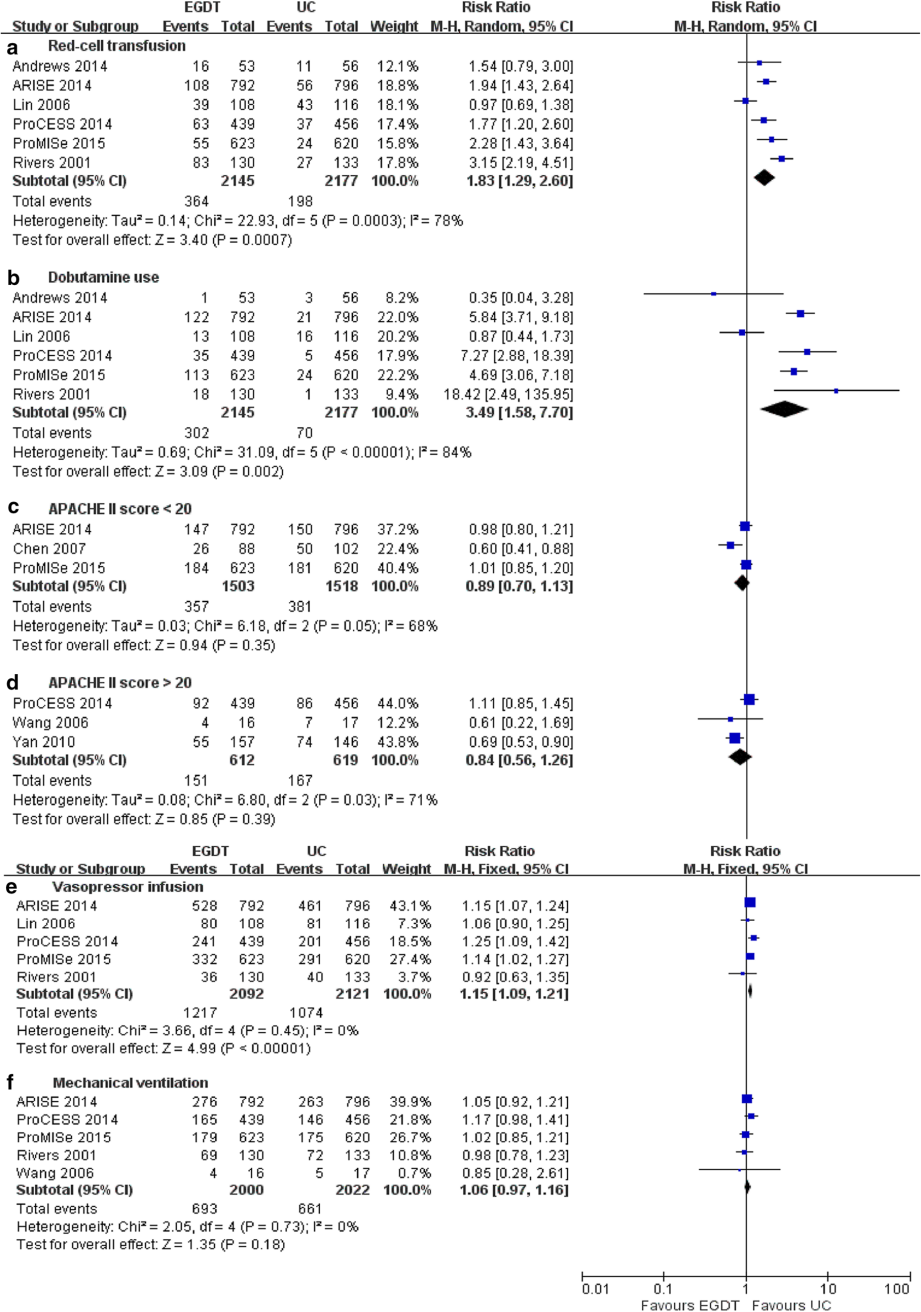
Forest plot showing EGDTvs. UC regarding red cell transfusions, dobutamine use, vasopressor infusion, and mechanical ventilation within the first 6 h and APACHE II score in patients with severe sepsis and septic shock. The severity of illness was reported in each study by the APACHE II score, and the data were presented as the mean ± SD. The distinction between higher and lower severity of illness was determined according to the mean APACHE II score in each study. APACHE II scores > or < 20 were reported in 3 trials. RR < 1.0 favours EGDT

The details of the interventions used in the included studies [4, 6–8, 13–18, 27–32] are shown in Table 2. These trials provided data comparing the secondary outcomes of EGDT and UC. As shown in Fig. 6, the pooled results showed that within the first 6 h, patients in the EGDT groups received greater total volumes of red cell transfusions (6 trials; RR = 1.83, 95% CI 1.29–2.60, *P* = 0.0007; *I*^2^ = 78%, *P* = 0.0003) [4, 6–8, 28, 31], more dobutamine (6 trials; RR = 3.49, 95% CI 1.58–7.70, *P* = 0.002; *I*^2^ = 84%, *P *< 0.0001) [4, 6–8, 28, 31], and more vasopressor infusions (5 trials; RR = 1.15, 95% CI 1.09–1.21, *P *< 0.0001; *I*^2^ = 0%, *P* = 0.45) [4, 6–8, 31] than UC patients, but no difference in the mechanical ventilation rate was found between the two groups (5 trials; RR = 1.06, 95% CI 0.97–1.16, *P* = 0.18; *I*^2^ = 0%, *P* = 0.73) [4, 6–8, 32]. The groups did not differ significantly regarding APACHE II scores > 20 and < 20 (RR = 0.84, 95% CI 0.56–1.26, *P* = 0.39; *I*^2^ = 71%, *P* = 0.03; and RR = 0.89, 95% CI 0.70–1.13, *P* = 0.35; *I*^2^ = 68%, *P* = 0.05, respectively).

**Table 2:** Details of the interventions used in the included studies

Study	Red cell transfusion within the first 6 h		Vasopressor infusion within the first 6 h		Dobutamine use within the first 6 h		Mechanical ventilation within the first 6 h		APACHE II score	
	EGDT	UC/LGT	EGDT	UC/LGT	EGDT	UC/LGT	EGDT	UC/LGT	EGDT	UC/LGT
Rivers 2001 [4]	83 (63.8%)	27 (20.3%)	36 (27.7%)	40 (30.1%)	18 (13.8%)	1 (0.8%)	69 (53.1%)	72 (54.1%)	21.4 ± 6.9	20.4 ± 7.4
Lin 2006 [31]	39 (36.1%)	43 (37.1%)	80 (74.1%)	81 (69.8%)	13 (12.0%)	16 (13.8%)	–	–	–	–
Wang 2006 [32]	–	–	–	–	–	–	4 (25.0%)	5 (29.4%)	28.0 ± 7.0	27.0 ± 6.0
Chen 2007 [29]	–	–	–	–	–	–	–	–	16.53 ± 6.87	16.68 ± 7.14
He 2007 [30]	–	–	–	–	–	–	–	–	–	–
Yan 2010 [27]	–	–	–	–	–	–	–	–	23.5 ± 5.7	21.8 ± 6.5
Andrews 2014 [28]	16 (30.2%)	11 (19.7%)	–	–	1 (1.9%)	3 (5.4%)	–	–	–	–
ARISE 2014 [7]	108 (13.6%)	56 (7.1%)	528 (66.7%)	461 (57.9%)	122 (15.4%)	21 (2.7%)	276 (34.8%)	263 (33.0%)	15.4 ± 6.5	15.8 ± 6.5
ProCESS 2014 [6]	63 (14.4%)	37 (8.1%)	241 (54.9%)	201 (44.1%)	35 (8.0%)	5 (1.1%)	165 (37.6%)	146 (32.0%)	20.8 ± 8.1	20.7 ± 7.5
ProMISe 2015 [8]	55 (8.8%)	24 (3.9%)	332 (53.3%)	291 (46.9%)	113 (18.1%)	24 (3.9%)	179 (28.7%)	175 (28.2%)	18.7 ± 7.1	18 ± 7.1
Jansen 2010 [14]	–	–	113 (63.8%)	119 (69.6%)	58 (32.8%)	69 (40.4%)	–	–	22.7 ± 9.1	23.6 ± 8.6
Jones 2010 [15]	5 (3.3%)	11 (7.3%)	113 (75.3%)	108 (73.5%)	8 (5.3%)	5 (3.3%)	39 (26.0%)	40 (26.7%)	–	–
Tian 2012 [17]	–	–	–	–	–	–	–	–	20.9 ± 8.6	17.8 ± 6.3
Yu 2013 [13]	5 (20.0%)	4 (16.0%)	20 (80.0%)	18 (72.0%)	1 (4%)	2 (8%)	19 (76%)	20 (80%)	17.91 ± 3.77	18.18 ± 6.01
Wang 2014 [18]	–	–	–	–	–	–	–	–	19.7 ± 3.1	20.9 ± 7.6
Lv 2015 [16]	–	–	–	–	–	–	–	–	–	–

The APACHE II score data are presented as the mean ± SD

EGDT, early goal-directed therapy; UC, usual care; LGT, lactate-guided therapy; APACHE II score, acute physiology and chronic health evaluation II score; –, no available

**Fig. 7:**
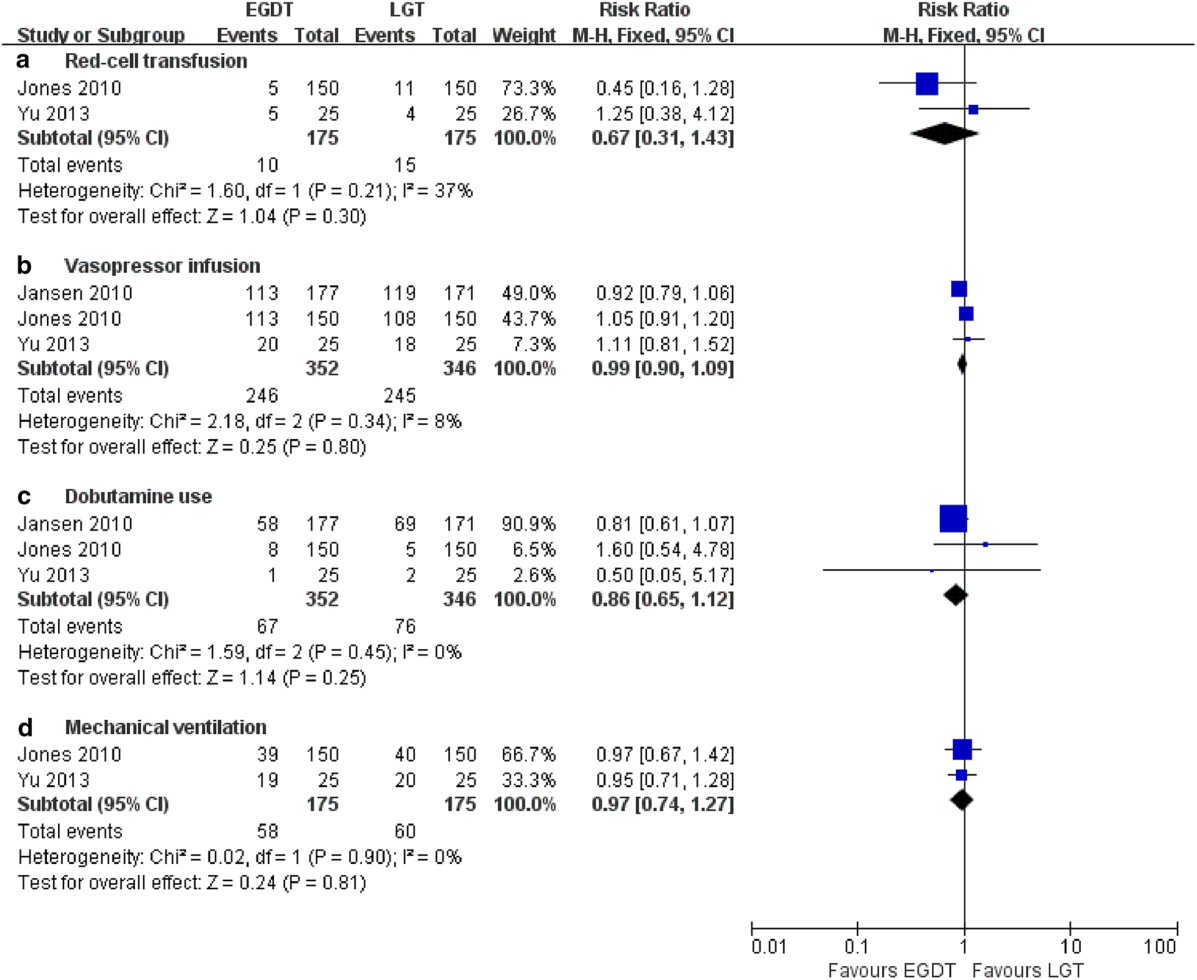
Forest plot showing EGDT vs. LGT regarding red cell transfusion, vasopressor infusion, dobutamine use, and mechanical ventilation within the first 6 h among patients with severe sepsis and septic shock. RR < 1.0 favours EGDT

As shown in Fig. 7, a total of 3 trials [13–15] provided information on the comparisons of EGDT and LGT regarding secondary outcomes. Red cell transfusions, vasopressor infusions, dobutamine use, and the mechanical ventilation rate did not significantly differ between the EGDT and LGT groups in the fixed-effects model (RR = 0.67, 95% CI 0.31–1.43, *P* = 0.30; *I*^2^ = 37%, *P* = 0.21; RR = 0.99, 95% CI 0.90–1.09, *P* = 0.80; *I*^2^ = 8%, *P* = 0.34; RR = 0.86, 95% CI 0.65–1.12, *P* = 0.25; *I*^2^ = 0%, *P* = 0.45; and RR = 0.97, 95% CI 0.74–1.27, *P* = 0.81; *I*^2^ = 0%, *P* = 0.90, respectively).

## Discussion

### Main findings

#### EGDT vs. UC

This meta-analysis showed that EGDT resulted in a 15% reduction in all-cause mortality compared with UC in adult patients with severe sepsis and septic shock, and this difference was particularly evident in the subgroup of UC patients with a mortality rate > 30%. The results also showed that EGDT had a significant mortality benefit prior to publication of the SSC2012.

Given these results, the efficacy of EGDT remains unclear, as the ProCESS, ARISE, and ProMISe trials [6–8] compared EGDT with UC and failed to show a mortality reduction. However, the results of these 3 trials [6–8] are highly controversial.

In this study, patients assigned to the EGDT group received more red cell transfusions, dobutamine and vasopressor infusions within the first 6 h than those assigned to UC. However, EGDT did not significantly differ from UC in the use of mechanical ventilation.

#### EGDT vs. LGT

We found that EGDT resulted in higher all-cause mortality compared with LGT regardless of whether the subgroup receiving LGT had a mortality rate > or < 30%. In otherwords, another strength of our findings was that LGT was found to decrease mortality compared with EGDT. Furthermore, use of red cell transfusions, dobutamine, vasopressor infusions, and mechanical ventilation did not differ between EGDT and LGT. Jansen et al. [14] reported that the hospital mortality of EGDT in ICU was 43.5%, and which of LGT was 33.9%. This study suggests that increased blood lactate levels have been associated with significant morbidity and mortality in ICU patients, and initial treatment aimed at reducing lactate levels has clinical benefit. It also indicated in patients with hyperlactatemia on ICU admission, lactate monitoring followed by targeted treatment significantly reduced ICU length of stay. However, monitoring itself cannot improve outcome; therefore, the therapeutic plan associated with the monitoring is equally important. The main differences in therapy between the two groups in the treatment period were the administration of more fluids and the increased use of vasodilators in patients assigned to the lactate group. In addition, Wang et al. [18] and Tian et al. [17] reported that LGT had a more lower mortality than EGDT in sepsis. However, we need multi-centre, large sample clinical trials and animal studies to explore the mechanism of LGT in the treatment of sepsis.

### Comparison with other studies

After the ProCESS, ARISE, and ProMISe trials [6–8] failed to show that EGDT decreased mortality in severe sepsis and septic shock, some studies [33–35] updated their meta-analyses to provide the latest and most convincing evidence regarding EGDT in sepsis. Similar to the findings of previous meta-analyses [33–35], our study found that EGDT was associated with lower mortality than UC, especially in more severe patients. We aimed to explain this difference in results by considering several factors. First, the mortality of the three well-known trials [6–8] was reported for UC patients with a mortality < 30% (Fig. 5). Consistent with the findings of our meta-analysis, EGDT has shown significant mortality benefits when the mortality of UC is > 30%. Second, we found that patients assigned to EGDT received more red cell transfusions, dobutamine and vasopressor infusions than patients in the UC groups. The SSC 2016 [9] recommended that red cell transfusion occur only when the haemoglobin concentration decreases to < 7.0 g/dl in adults in the absence of extenuating circumstances, such as myocardial ischemia, severe hypoxemia, or acute haemorrhage (strong recommendation, high quality of evidence). In addition, EGDT patients received longer mechanical ventilation, but this difference was not significant. In conclusion, EGDT may lead to more positive outcomes than UC. The SSC 2016 also stated that early effective fluid resuscitation is critical to stabilization of sepsis-induced tissue hypoperfusion or septic shock [9]. Third, Levy [36] raised the question of how ‘usual’ care and ‘real-world’ care are defined. The treatment provided with UC has increasingly trended towards that proposed by Rivers [4]. In Fig. 3, the RRs gradually neared 1 when arranging the trials in chronological order. This finding indicates that UC treatment gradually changed over time.

How do our study results compare with those of previous meta-analyses? Gu et al. [34] analysed only four randomized trials; thus, their results may be false-positive or may not be as robust or reliable. Lu et al. [35] did not conduct detailed or comprehensive subgroup analyses to assess the differences between EGDT and LGT and included only 13 studies. Finally, another study by Gu et al. [33] included studies from a different time period.

In sepsis, a more controversial issue is the index used to reflect tissue oxygen delivery. The SSC 2012 recommended using Scvo_2_ to assess the balance between tissue oxygen delivery and consumption [37]. However, there are still some challenges to identifying patients with sepsis, and the largest roadblocks to overcome in implementing Scvo_2_ include time, expertise, adherence, and need for specialized equipment [38]. Currently, studies have found that blood lactate concentration could be used to reflect the restoration of oxygen delivery in resuscitation and have confirmed that lactate monitoring is useful [39]. However, the optimal measurement choice in different situations remains unclear. The ProCESS, ARISE, and ProMISe trials were unable to recommend using Scvo_2_ from their findings. In addition, the SSC2016 recommended viewing these patients as experiencing a medical emergency requiring urgent assessment and treatment and suggested guiding resuscitation by normalizing lactate inpatients with elevated lactate levels, as these levels may serve as a marker of tissue hypoperfusion [9]. As LGT is non-invasive, when the same monitoring effort is expended, we can recommend LGT as the better option.

### Strengths and limitations

This meta-analysis included 10 RCTs that systematically compared EGDT with UC in 5051 patients, and the results showed a 15% reduction in all-cause mortality in adult patients with severe sepsis and septic shock. Another strength of this study was the finding that LGT may have a greater mortality benefit than EGDT.

Our study has several limitations. First, seven trials [17, 18, 25, 27, 29, 30, 32] had an unclear risk of bias. Second, the effect of region-specific variations in clinical practice could not be assessed in our meta-analysis.

## Conclusions

Our meta-analysis showed that a 6-h EGDT protocol for patients with severe sepsis or septic shock resulted in lower mortality than UC, especially in severely ill patients. The most likely reason for this finding is that patients assigned to EGDT received more red cell transfusions, dobutamine, and vasopressor infusions within the first 6 h. Compared with EGDT, LGT was associated with lower mortality, despite the lack of differences in the use of red cell transfusions, dobutamine, vasopressor infusion, and mechanical ventilation. However, in the future, we look forward to an updated meta-analysis including more high-quality RCTs mainly related to LGT versus EGDT and taking into account red cell transfusions, vasopressor infusions, dobutamine use, and the use of mechanical ventilation within the first 6 h, which may provide more precise guidance for clinicians in the treatment of sepsis.

## Additional file

**Additional file 1.** PRISMA 2009 checklist.

## Abbreviations

EGDT: early goal-directed therapy
LGT: lactate-guided therapy
UC: usual care
RCT: randomized controlled trials
ED: emergency department
Scvo_2_: systemic central venous oxygen saturation
SSC: Surviving Sepsis Campaign
APACHE II: Acute Physiology and Chronic Health Evaluation II
PRISMA: preferred reporting items for systematic reviews and meta-analyses
RR: relative risk
CI: confidence interval
M–H: Mantel–Haenszel
